# Distributed Relative Localization Algorithms for Multi-Robot Networks: A Survey

**DOI:** 10.3390/s23052399

**Published:** 2023-02-21

**Authors:** Shuo Wang, Yongcai Wang, Deying Li, Qianchuan Zhao

**Affiliations:** 1 School of Information, Renmin University of China, Beijing 100872, China; 2 Metaverse Research Center, Renmin University of China, Beijing 100872, China; 3 Department of Automation, Tsinghua University, Beijing 100084, China

**Keywords:** distributed localization, relative localization, multi-robot systems, distributed SLAM

## Abstract

For a network of robots working in a specific environment, relative localization among robots is the basis for accomplishing various upper-level tasks. To avoid the latency and fragility of long-range or multi-hop communication, distributed relative localization algorithms, in which robots take local measurements and calculate localizations and poses relative to their neighbors distributively, are highly desired. Distributed relative localization has the advantages of a low communication burden and better system robustness but encounters challenges in the distributed algorithm design, communication protocol design, local network organization, etc. This paper presents a detailed survey of the key methodologies designed for distributed relative localization for robot networks. We classify the distributed localization algorithms regarding to the types of measurements, i.e., distance-based, bearing-based, and multiple-measurement-fusion-based. The detailed design methodologies, advantages, drawbacks, and application scenarios of different distributed localization algorithms are introduced and summarized. Then, the research works that support distributed localization, including local network organization, communication efficiency, and the robustness of distributed localization algorithms, are surveyed. Finally, popular simulation platforms are summarized and compared in order to facilitate future research and experiments on distributed relative localization algorithms.

## 1. Introduction

With the development of robots, drones, unmanned vehicles, edge devices, and various sensor technologies, networks formed by multiple robots have great potential in various applications. The robots form a network with each other through communication, observation, and collaboration, and this can play an important role in exploration, rescue, firefighting, transportation, and many other tasks [1]. The robots’ ability to autonomously localize themselves is the basis for accomplishing these tasks [2]. Therefore, the precise localization of a swarm of multiple robots has attracted great interest.

The global navigation satellite system (GNSS) has made rapid progress in recent years, and several reliable systems have emerged, such as GPS, Beidou, GLONASS, and Galileo. GNSS has been widely used for localization [3], and can give the absolute locations for robots. The RTK (real-time kinematic) technique can help GNSS localization reach centimeter-level accuracy [4,5]. This provides great help for autonomous driving and autonomous navigation.

However, GNSS becomes unreliable or unavailable in many harsh environments, such as indoors, underground, outer space, and battlefields. Relative localization is an alternative way to localize the robots in a network using the measurements between the robots without information from outside [6]. Relative localization algorithms can be divided into centralized and distributed (or decentralized).

In centralized systems [7,8], all the robots report their data to a central server. The central server then processes the computational tasks and returns the localization results to all the robots. In this centralized manner, the robots play the role of sensors in the localization task and do not take full advantage of their computational capabilities. In addition, the central node must bear huge computational and communication costs and is likely to incur latency, which is unsatisfying for robots with high real-time requirements.

In distributed relative localization [9,10], each robot calculates its location relative to neighbors, and there is no central station in the swarm. Each robot has only local information or partial information obtained by communication with neighbors; usually, no global information is available. Note that the distributed relative localization algorithms do not only come from partitioning the centralized algorithms but often require additional design for sensing, communication, networking, etc.

At first, distributed relative localization of robots relies on relative observations when no global localization information is available. Observations of robots can be divided into two categories: (1) observations between robots and (2) observations of the environmental features. Observations between robots include the range, angle, and other measurements that can estimate relative states. Environmental measurement information includes extracting environmental feature points, estimating the location of environmental landmarks, and creating environmental maps using sensors, such as cameras and LiDAR.

Environmental information can also generate observations between robots. For example, inter-robot loop closure can be detected using existing maps or landmarks. As the front end of the localization system, the robot-sensing and data-processing modules have important impacts on the communication and solution modules in the distributed relative localization framework.

Secondly, in distributed relative localization, the neighborhood’s state information needs to be transmitted through the communication module. Each robot has a limited range of observation in the network [11]. Each robot needs to communicate with its neighbors to obtain more information, thus, localizing itself collaboratively. In this process, the robot node needs to communicate with its neighbors for information to flow in the network. Efficient communication allows the robot to receive timely updates from its neighbors, thus, avoiding errors caused by packet loss or communication delays [12].

Thirdly, distributed computation is the core part of distributed relative localization in multi-robot networks. Each node calculates its relative locations with the neighbors, and the robots need to synchronize their estimates to generate consistent relative localization results in the network. This progress generally involves state propagation and state iteration.

For the above reasons, distributed relative localization systems have many new challenges compared to centralized network localization. The first challenge is that it is necessary to design distributed and efficient communication protocols. The second challenge is that each node constructs a local graph with its neighbors, which is likely to be sparse. The localizability in sparse networks is hardly satisfied compared with in dense networks [13]. More importantly, each robot must calculate its coordinates without global information and instead through neighborhood communication and state iteration. Distributed location algorithms are an emerging research topic in the field of robotics. How to design a distributed relative localization algorithm with high precision, high efficiency, and high robustness is still an open research problem.

We categorize and discuss the literature in this survey according to the measurement method because the algorithm design for localization is tightly coupled with the sensor measurement information. Specifically, we divided the distributed relative positioning into three categories: distance-based localization, bearing-based localization, and multiple measurement fusion. In each type, different algorithms and system designs are analyzed. In addition, we find that simultaneous localization and mapping, as an emerging research topic in recent years, also involves a great deal of research on localization algorithms.

This survey investigates and analyzes the distributed multi-robot simultaneous localization and mapping algorithms. This survey also discusses, in depth, the key issues of distributed positioning, including local network organization, communication, robustness, challenges of current research, future trends, and experiment platforms.

To the best of our knowledge, this work is the first thorough survey on distributed relative localization algorithms for multi-robot networks. The key contributions are as follows:

- This work summarizes the distributed multi-robot network relative localization methods and classifies various methods according to their design methodologies and types of measurements. A detailed introduction is presented for each type of distributed relative localization algorithm. The advantages and limitations of each type of distributed relative localization algorithm are analyzed and compared.
- Key problems in distributed localization algorithms, namely local subnetwork organization, communication efficiency, and the robustness of distributed localization, are investigated.
- The popular simulation experiment platforms are investigated, and the characteristics of each platform are analyzed from different aspects, which can be a reference for researchers conducting simulation experiments.

## 2. Related Reviews

Distributed localization algorithms in robot networks have attracted great interest from researchers. Although there are some surveys of related topics, an overview of distributed relative localization algorithms is still needed. Yuan et al. [14] surveyed the localization methods for a single robot and conducted detailed research on both single-sensor and multi-sensor fusion solutions. Çaşka et al. [15] introduced a summary of the various UAV/UGV collaboration frameworks and decentralized sensor fusion methods. However, they presented little regarding distributed methods.

Chowdhury et al. [16] presented the localization methods in wireless sensor networks without a detailed introduction of distributed methods. Khelifi et al. [17] provided a detailed classification and comparison of centralized and distributed approaches. However, the study was based on traditional IoT sensors, and there was no scheme for multi-sensor fusion and optimization. Wang et al. [18] surveyed the UWB-based localization algorithms, which means that only distance measurements were available in the network. Sun et al. [19] conducted a survey of graph realization algorithms, which are also based on distance measurements.

With the development of simultaneous localization and mapping technology in recent years, Gupta et al. [20] summarized the scheme of multi-robot collaborative SLAM. However, fully distributed SLAM algorithms are still relatively sparse in the literature. We compared our work with related survey works, and the results are shown in Table 1. To the best of our knowledge, this work is the first thorough survey of the distributed relative localization algorithms in multi-robot networks. This survey not only introduces the localization algorithm design but also covers different observations, communication schemes, local graphs, experimental platforms, etc.

**Figure 1:**
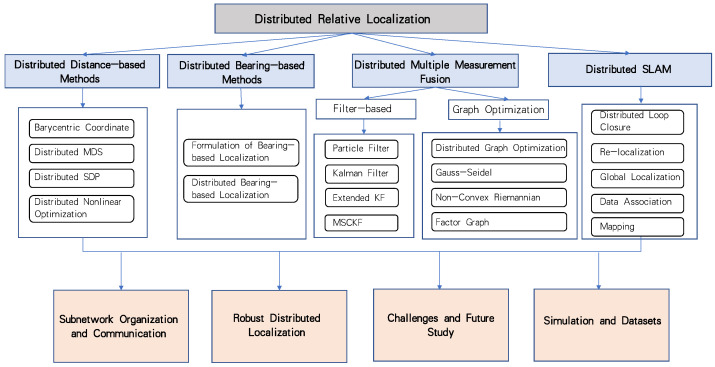
The main content of this survey.

The rest of this paper is organized as follows. Section 3 to Section 6 introduce distributed relative localization algorithms using distance measurements, bearing measurements, fusion methods, and SLAM methods, respectively. Section 7.1 introduces the local network organization methods for distributed localization algorithms. Section 7.2 introduces the studies on the communication efficiency for distributed localization. The robustness of localization algorithms is presented in Section 8. Section 10 presents the current popular simulation platforms. Finally, a brief conclusion is given in Section 11. We summarize the main content of this survey in Figure 1.

First, we classify the algorithms according to the specific measurement information, i.e., the distance, bearing and multiple measurement fusion. We present the main features of the algorithms and analyze the application scenarios and shortcomings of these algorithms in the following sections.

## 3. Distance-Based Distributed Relative Localization Methods

Distance measurement is the most common measurement information in IoT and robotic networks. Distance measurement can be obtained by sensors, such as UWB, Bluetooth, WIFI, and RFID. Common methods include RSS (received signal strength), TOA (time of arrival), and TDOA (time difference of arrival) [19]. The distance measurements are characterized light weight, low power consumption, and high frequency. Therefore, distance-based relative localization is popular [22]. Distributed localization algorithms can be further classified into two categories:

1. Barycentric coordinate-based methods, which present a linear representation of the localization problem and derive naturally distributed iterative algorithms running at distributed nodes that can guarantee converging to the correct states under specific conditions.
2. Distributed algorithms developed from their centralized versions, which include: (i) distributed multi-dimensional scaling (MDS), (ii) distributed semi-definite programming (SDP), and (iii) distributed nonlinear optimization.

We introduce related algorithms in the following subsections.

### 3.1. Barycentric Coordinate-Based Algorithms

The barycentric coordinates are a geometric representation, and they were proposed by August Ferdinand Möbius in 1827. They are used to mark the position of a point relative to other points. Barycentric coordinates are widely used in network-localization methods based on ranging information [23].

#### 3.1.1. Formulation of Barycentric Distributed Localization

To explain this representation, recall that four points $j,k,l,h\in R^{3}$ and their three-dimensional Euclidean coordinates are $p_{j},p_{k},p_{l},p_{h}$. If the coordinates of point *i* satisfy the following equation:

(1) $$a\left(t\right)=\left\{\begin{array}{c} p_{i}=a_{ij}p_{j}+a_{ik}p_{k}+a_{il}p_{l}+a_{ih}p_{h}\\ a_{ij}+a_{ik}+a_{il}+a_{ih}=1 \end{array}\right.$$

then $\left\{a_{ij},a_{ik},a_{il},a_{ih}\right\}$ are the barycentric coordinates of point *i* with respect to point j,k,l,h.

Khan et al. [24] proposed DILOC, a barycentric coordinate-based approach for distributed network localization. DILOC uses the barycentric coordinates of a node with respect to its neighbors. Specifically, the barycentric coordinates of node *l*, $a_{lk}$, with respect to its neighbor node *k*, are given by:

(2) $$a_{lk}=\frac{A_{\left\{l\right\}\cup \Theta_{l}\setminus \left\{k\right\}}}{A_{\Theta_{l}}}$$

where $\Theta_{l}$ is the triangulation set of node *l* and $A_{\Theta_{l}}$ is the generalized volume (3D) or area (2D) of $\Theta_{l}$’s convex hull. The barycentric coordinates are computed distributedly using the Cayley–Menger determinant [25]. In particular, the localization of an agent can be realized from a set of m+1 anchors in an *m*-dimensional Euclidean space. Since each node has limited communication and perception distance, the localization of agents is updated iteratively by:

(3) $$c_{l}\left(t+1\right)=\left\{\begin{array}{c} c_{l}\left(t\right),l\;is\;an\;anchor\\ \sum_{k\in \Theta_{l}}a_{lk}c_{k}\left(t\right),l\;is\;not\;an\;anchor \end{array}\right.$$

This formulation converts the non-linear localization problem to a linear state iteration problem at distributed nodes according to the barycentric coordinate theory. Several works followed this idea [13,23,26,27,28,29,30]. However, the main challenges for these barycentric-based localizations are: (1) the noises of distance measurement are not well handled and (2) all the nodes to be localized must lie strictly inside the convex hull spanned by the anchors.

#### 3.1.2. Dealing with Convex Hull Constraints and Noises

To overcome these obstacles, Diao et al. [27] developed an approach based on general barycentric coordinates, including positive and negative values. The barycenter node does not need to be located in the convex hull formed by its neighbors. This method calculates the barycentric coordinates of each group of d+1 (*d* is the space dimension) neighbors by traversing the combination of the neighbors. The final barycentric coordinates result from the fusion of these weighted coordinates. This algorithm utilizes the coordinates of all neighbors—that is, more abundant local information. Richardson Iteration is used in the solution process, and the parameter values are given, which ensures global convergence and accelerates the iteration speed.

More recently, Xia et al. [29] presented an analytic solution to calculate the 3D barycentric coordinates for agents outside the convex hull formed by anchors, which can be applied in large scale scenes compared with DILOC [24]. They proposed symbol parameters:

(4) $$\sigma_{01}|\omega_{01}|+\sigma_{02}|\omega_{02}|+\sigma_{03}|\omega_{03}|+\sigma_{04}\left|\omega_{04}\right|=1$$

where the symbol parameter $\sigma_{0i}$ is −1 or 1 and $\omega_{0i}$ is the barycentric coordinate. Although there are, at most, 15 possibilities for the selection of parameters, the above equation is not strictly true considering the error in the real world. Therefore, paper [29] turned to solving an optimization problem to determine the symbol:

(5) $$\begin{array}{cc} \; & Minimize:(\sigma_{01}|\omega_{01}|+\sigma_{02}|\omega_{02}|+\sigma_{03}|\omega_{03}|+\sigma_{04}|\omega_{04}{\left|-1\right)}^{2}\\ \; & Subject\;to:\sigma_{0i}=1\;or\;\sigma_{0i}=-1 \end{array}$$

Agents in the network can utilize the Jacobian under-relaxation iteration (JURI) algorithm to solve the localization problem under noisy measurements. The traditional Jacobian iterative is carried out by $p_{n}^{\left(k+1\right)}=D^{-1}\left(L+U\right)p_{n}^{\left(k\right)}+D^{-1}b$, where $p_{n}^{\left(k+1\right)}$ and $p_{n}^{\left(k\right)}$ are the results of the k+1 iteration and the *k* iteration, respectively. The author introduced a relation parameter $\alpha \in \left(0,1\right]$ for anti-disturbance and formed JURI:

(6) $$p_{n}^{\left(k+1\right)}=\left(1-\alpha \right)p_{n}^{k}+\alpha \left(D^{-1}\left(L+U\right)p_{n}^{\left(k\right)}+D^{-1}b\right)$$

These developments make the solution of barycentric coordinates not restricted by a convex hull and able to deal with the measurement noises as well. Another recent work [13] exploited the node localizability problem in barycentric localization. Ping et al. [13] showed that, when unlocalizable nodes participate in the iterative location updating, the localization results may converge incorrectly. For detecting localizable nodes, in [13], Ping et al. presented a centralized iterative maximum flow algorithm. In their more recent work [31], Ping et al. presented a negative edge inference method and a distributed algorithm for localizable node detection, which is suitable to be used in distributed barycentric localization methods.

#### 3.1.3. Communication Problems for Barycentric Distributed Localization

Other problems to be solved are communication delay and packet loss. Huang et al. [28] developed a synchronous communication protocol taking communication delays and packet loss into account. Each agent maintains a cache to store their neighbors’ latest iteration (marked as *k*) information. Some packets will arrive late due to communication delay or packet loss. The agent will compare the $k^{\prime }-iteration$ data newly arrived with the storage and will update the cache when and only when $k^{\prime }>k$. The algorithm’s convergence is guaranteed with bounded communication delays and packet loss.

In addition to the direct calculation of barycentric coordinates, we can utilize the fact that a congruent framework of the subnetwork consisting of the node and its neighbors has the same barycentric coordinates in any dimensional space. Based on this technique, Han et al. used a linear equation related to a signed Laplacian to describe the geometric constraints of the whole network [26]. Each node forms a subnetwork and calls for a multidimensional scaling (MDS) method to construct a congruent framework, satisfying that $d_{ij}=∥p_{i}-p_{j}∥$ for any node i,j in the subnetwork. During the process, a linear equation is solved through the distributed linear iterative algorithm to obtain its global localization.

### 3.2. Distributed Algorithms Transformed from Centralized Algorithms

Unlike barycentric coordinate-based distributed localization, which has a naturally distributed formulation, many distributed relative localization algorithms using distance information are transformed from traditionally centralized localization algorithms. This subsection mainly introduces the distributed version of multi-dimensional scaling (MDS), semi-definite programming (SDP), and distributed nonlinear optimization methods.

#### 3.2.1. Distributed Multi-Dimensional Scaling

MDS is one of the dimension-reduction techniques that can change the dimensions of data while maintaining the information. MDS is widely used in wireless sensor networks (WSN) and the internet of things (IoT) to localize agents. For localization, MDS methods require pairwise Euclidean distances of nodes in the network. The MDS algorithm was originally designed as a centralized positioning method [32], in which the computation center collects all the measurements and performs the algorithm.

MDS needs to perform the deposition of N×N matrix when the network has *N* nodes. When *N* is large, the algorithm suffers from a high computational burden. For applications in robot networks, it is necessary to design a distributed algorithm to distribute the computation to each robot to speed up the algorithm. Distributed MDS algorithms are usually divided into three steps:

- Step 1: Divide the network into sub-networks (clusters). In the cluster, pairwise distances are needed.
  In distributed systems, each agent in the network runs the division algorithm individually and asynchronously without centralized control and scheduling. Generally, we can divide the network according to the characteristics of the graph nodes. The simplest way is to divide the nodes according to their degree centrality [9,33]. First, for each node *i* in the network, construct the set $S_{i}$ consisting of all the direct neighbors of node *i*. Then, update $S_{i}$ by $S_{i}=S_{i}\cup S_{j}$ for $\forall j\in S_{i}$. Search the largest cardinality group in the network and set it as a cluster. Repeat the process in the last part of the network until the network is divided.
- Step 2: Perform the MDS-based algorithm in each cluster to calculate the relative coordinates. This step constructs the accurate local graph.
  We can conduct the classic MDS method to calculate the relative coordinates in the cluster. While to reduce the computational complexity, the improved MDS, MDS-MAP [34], is deployed in many papers [26,33,35]. The MDS-MAP algorithm uses the Floyd algorithm in each cluster to fill in the missing range measurements, thus, reducing the range error caused by too many hops.
  However, the method does not address the problem of errors in the measurement itself. When the measurement error is significant, the matrix in the MDS algorithm may not be able to perform the eigenvalue decomposition. The operations on linear equations and matrix decomposition in MDS are susceptible to measurement errors. In paper [26], the authors use singular value decomposition instead of eigenvalue decomposition to deal with the connection matrix and compute the relative coordinates matrix *Z*. Then, a more accurate matrix *Z* is obtained by minimizing $∥B-Z^{T}{Z∥}_{2}^{2}$, where *B* can be derived from the matrix composed of the square of the distances.
- Step 3: Merge all the local graphs into a global network based on the common nodes in each local graph. This is a key step in the algorithm, especially for large networks [36,37].
  The traditional method uses the overlapping edges between subgraphs for merging [38], but this method is prone to ambiguity. Kim et al. [35] add a distance constraint to the splicing process, i.e., they choose the network that does not generate new edges as the result.
  In fact, the uniqueness of subgraph merging can be determined: Two individual maps can be uniquely merged in *m*-dimensional space if and only if at least m+1 common nodes exist. Dan et al. [33] propose that merging remains unique when the number of common nodes is less than m+1; however, some specific edges exist. Based on these conditions, they design corresponding merging strategies that guarantee uniqueness.

### 3.3. Distributed Semi Definite Programming

Semi-Definite Programming is a convex optimization method. SDP is widely used in wireless sensor networks for localization [39,40], and also in robotic networks using distance measurements. In SDP, multi-robot localization can be expressed in the following form [39]. Let $X=[x_{1},x_{2},\ldots ,x_{n}]$ contain *n* coordinates to be localized. The constraints of the problem can be written as:

(7) $$\begin{array}{cc} \; & {\left(e_{i}-e_{j}\right)}^{T}Y\left(e_{i}-e_{j}\right)=d_{ij}^{2}\\ \; & {\left(\begin{array}{c} a_{k}\\ -e_{j} \end{array}\right)}^{T}\left(\begin{array}{cc} I_{2} & X\\ X^{T} & Y \end{array}\right)\left(\begin{array}{c} a_{k}\\ e_{j} \end{array}\right)=d_{jk}^{2}\\ \; & Y=X^{T}X \end{array}$$

where $d_{ij}$ is the distance between *i* and *j*, $a_{k}$ is the coordinates of anchor *k*, and $e_{i}$ is a vector with all zeros except its *i*th entry.

The traditional SDP localization algorithm [39] is to perform relaxation on (7). The square matrix *Y* in (7) is changed to $Y⪰X_{T}X$. Then, *Z* is written as:

(8) $$Z=\left(\begin{array}{cc} I & X\\ X_{T} & Y \end{array}\right)⪰0$$

Replace *Y* in (7) by *Z* and then we can obtain a standard SDP problem.

The framework of the distributed SDP algorithm is similar to that of the MDS algorithm (Section 3.2.1). The distributed SDP algorithm consists of three steps: subgraph division, local computation, and subgraph stitching. The computation cost of the SDP algorithm is $O(n^{3.5})$ so the subgraph division is usually applied to reduce the computation cost, especially in the large-scale networks.

Li et al. [41] propose a partition algorithm to divide the graph according to the importance index of each node. In each cluster, the node with the largest importance index is selected as a cluster head. In each cluster, a local semidefinite programming(LSDP) is carried out distributedly on each node to compute a local map centered by the cluster head. Then, the local maps are merged into a global map by solving a least squares optimization problem.

For the local computation step, the SDP-based algorithms are applied to compute the local structure of each robot node. In the method proposed by Biswas et al. [42], they use a semidefinite programming relaxation and gradient search to localize the local subgraphs, and complete the distance matrix with SDP. In order to solve the problem of incorrect localization of some nodes in some subgraphs, this method will use the nodes that have been precisely positioned as anchors for assistance, making up for the lack of anchors.

In the algorithm proposed by Liu et al. [43], the 3D relative position estimation is proved to be a constrained optimization and the relative CRLB is obtained as the constrained CRLB. A weighted SDP algorithm is utilized to obtain the local geometry structure and then the distributed geometry-based merging algorithm merges the local structures into a global graph.

### 3.4. Distributed Nonlinear Optimization

The relative localization problem can be transformed into an optimization problem in essence. The optimization algorithm sets an initial estimate, and then minimizes the error between the estimation and the ground truth of the measurement [44]. Let $X=[x_{1},x_{2},\ldots ,x_{n}]$ denotes *n* coordinates to be localized and $d_{ij}$ represents the distance measurement between robot node *i* and *j*. The error function of robot *i* to be minimized is:

(9) $$F=\sum_{j\in N(i)}\left(|\right|x_{i}-x_{j}\left|\right|-d_{ij}{)}^{2}$$

where $\left|\right|x_{i}-x_{j}\left|\right|$ is the distance predicted by the current states and N(i) is the neighbor of node *i*. In centralized version, the error functions of all nodes are added to obtain the error function of the whole network. Then, the optimal robot coordinates are obtained through nonlinear optimization algorithm [45].

**Figure 2:**
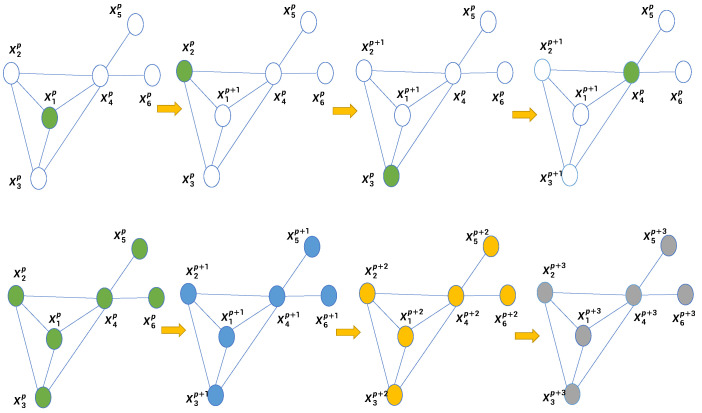
Sequential iteration (**top**) and parallel iteration (**bottom**). $X_{i}^{p}$ denotes the state of the *i*-th node after p−th iteration.

Cheng et al. [11] proposed a distributed Gaussian Newton method for designing distributed graph optimization algorithms. There are *m* known anchors and *n* sensors with unknown positions in the network. Each node builds a nonlinear optimization problem based on local measurements and the current position of its neighbors and iteratively solves it. The paper provides two types of iterations: *sequential iteration* and *parallel iteration* (Figure 2). Sequential iteration means that all nodes are solved sequentially, which leads to higher accuracy but slower iteration speed. In parallel iteration, all nodes start computing at the same time, which results in a faster iteration speed with some loss of accuracy.

Bejar et al. [46] used consensus to derive a distributed version of the Gauss–Newton method by exploiting the inherent block-wise structure of the network in the same spirit. The algorithm does not require knowledge about the network topology or size, and thus it is more robust and scalable. Priyantha et al. [47] proposed the concept of fold-freedom to explore the anchor-free distributed localization algorithm. The algorithm seeks a “fold-free” configuration which is locally distorted version of the groundtruth. The nodes run a force-based relaxation procedure taking care to not seriously violate fold-freedom while minimizing the error. The result then is a correct solution.

In summary, distance-based methods can provide accurate 3D positions of the robot with lightweight ranging devices. Table 2 summarizes and compares the distance based distributed localization algorithms. Barycentric based methods generally have slow convergence speed and are sensitive to ranging noises. Distributed MDS and distributed SDP generally require the distance graph to be dense [48]. Distributed nonlinear optimization can tolerate sparseness in some extend and generally have high convergence speed. Distributed nonlinear optimization can also be used when multiple kinds of observations are used. Therefore, distributed nonlinear optimization is widely applied in solving multi-robot distributed localization problems.

On the other hand, solving for the robot’s pose or sensor orientation is also indispensable in some scenarios. Although some approaches install multiple ranging nodes on the robot device to estimate the relative angle [49], the deployment of the device and the algorithm design are too complex and do not guarantee good accuracy. Therefore, angular sensors, such as cameras are particularly necessary.

## 4. Bearing-Based Distributed Relative Localization Methods

Robots can measure bearing information among robots through Cameras, LIDAR, sonar and certain Bluetooth devices. Therefore, bearing-based relative localization methods are widely exploited.

### 4.1. Formulation of Bearing-Based Localization

The camera is widely equipped on smart unmanned devices as a low-cost, light-weighted device. The camera can either obtain angle/bearing measurement between robots from object detection through neural network or pattern recognition, or it can extract environmental feature points [51]. LIDAR, sonar, and some Bluetooth can also be used to obtain the bearing, which can be used in specific environments [49]. The bearing-based relative localization methods share the following model.

In 2D networks, the state of robot *i* can be defined as $X_{i}=\left\{x_{i},y_{i},\eta_{i}\right\}$. The first two represent two-dimensional coordinates, and $\eta_{i}$ is the rotation. The bearing measurement between robots *i* and *j* is:

(10) $$Z_{ij}=\arctan\left(\frac{y_{j}-y_{i}}{x_{j}-x_{i}}\right)-\eta_{i}+\varepsilon^{i}$$

where $\varepsilon^{i}$ is modeled as the zero-mean Gaussian noise.

The traditional method of estimating relative rotations between robots using environmental feature points acquired by the camera is epipolar geometry [52]. When there is a sufficient range of overlap between the two robots’ fields of view, the two images can be obtained with a sufficient number of matched pairs of feature points. The epipolar geometry approach can solve the relative rotation between the two robots.

### 4.2. Distributed Bearing-Based Localization

Leonardos et al. [49] proposed a method that provides good accuracy. The paper uses the matched environmental feature points as external auxiliary points and the robot nodes to form a large-scale network topology graph. A distributed Riemann gradient descent algorithm is implemented in the graph to solve for the robots’ states. It is experimentally demonstrated that the method can be more accurate than the vision-based epipolar geometry method.

In fact, the bearing-only method can also be expressed and solved using the barycentric coordinate method. Cao et al. [30] proposed a bearing-only approach to estimate the relative pose. The barycentric coordinates of the nodes are first calculated by local goniometry and scale factors. Then, using the conclusion from the paper [49] that two mutually angular robots can recover the rotation matrix between them, the rotation and translation states of the final robot are obtained.

An important issue in bearing-only systems is to estimate the distance between robots. Yang et al. [53] introduced that the distance between the observed objects after bearing measurements can be modeled as a Gaussian distribution. When a robot can measure the relative angle of another robot (active measurement) or be measured by another robot (passive measurement), the local different distance probability models are fused with data by Chernoff Fusion to obtain an estimate of the final coordinates. However, the experiments find that the method has significant errors in measuring multiple hops.

Due to the singularity of bearing measurement information, the network suffers from the problem of localizability, which is the fundamental research problem of network localization. Fang et al. [54] proposed an angle-displacement rigidity theory to determine whether a set of angle and displacement constraints can uniquely localize a 3D network. Based on the angle-displacement rigidity theory, they designed a distributed localization algorithm protocol to estimate the locations of all nodes in a network globally.

Bearing-only methods suffer from many limitations, such as limited field of view, measurement range, occlusion between agents, and error distribution. Although these problems can be avoided in some cases by maintaining bearing rigidity [55], they are still challenges to be solved in fully distributed systems.

Both distance-only and bearing-only methods use single kinds of measurement, which is prone to errors when measurements are sparse and may encounter unlocalizability problems. Constructing multiple constraints using multiple sensors to achieve localization estimation for robotic swarms often leads to more stable and accurate results.

## 5. Multiple Measurement Fusion

More reliable and accurate robot localization can generally be achieved through multi-sensor fusion [56]. The sensors can cooperate to make up for each sensor’s defects. Recently, robot localization solutions have increasingly been using multi-sensor fusion architectures. We will introduce the framework for multi-sensor fusion in this section. The multiple sensors mainly provide distance, bearing, and environmental information. From fusion methodologies, sensor fusion methods can be mainly classified into (1) filter-based methods and (2) graph-optimization methods.

### 5.1. Filter-Based Multi-Sensor Fusion and Localization

Filtering methods have been widely applied in robot state estimation. The traditional filters used for state estimation are mainly the Bayesian filter, particle filter, Kalman filter, etc. [57]. In this subsection, we first introduce the methods based on a particle filter, and then present the form of Kalman filter and the relative localization algorithms based on a Kalman filter in detail.

#### 5.1.1. Particle Filter and Relative Localization

A particle filter was first introduced to solve the nonlinear Bayesian filter problem and has become a mature algorithm in the field of robotics [58]. Particle filters are widely used in location problems with moving robots as they provide optimal localization results for robots. In the particle filter, the motion model at time *t* can be described as:

(11) $$\begin{array}{c} X_{t}=f_{t}\left(X_{t-1},\delta_{t}\right)\\ Y_{t}=h_{t}\left(X_{t},\gamma_{t}\right) \end{array}$$

where $X_{t}$ is the predicted value of the position and $Y_{t}$ is the observed value. $\delta_{t}$ and $\gamma_{t}$ represent the state transition and observation noise, respectively.

The main idea of the solution is sampling. We can sample particles forming a set ${\left\{X_{t}^{\left(i\right)},W_{t}^{\left(i\right)}\right\}}_{i=1,\ldots ,N}$, where $X_{t}^{\left(i\right)}$ represents the particle and $W_{t}^{\left(i\right)}$ is the corresponding weight. The localization result is expressed as:

(12) $$E\left[f\left(X_{t}\right)\right]=\int f\left(X_{t}\right)p\left(X_{t}|Y_{1:t}\right)dX_{t}$$

where f(·) is the mapping of random variables to robot coordinates.

In different application scenarios, the process of a particle filter is also different. In paper [59], each node locally performs the needed calculations for the particle, including initialization, prediction, measurement update, resampling, and positioning. Once a sensor has obtained the initial position estimate, the Markov-chain Monte Carlo (MCMC) method is applied on each node to reduce the estimation error and propagation error in a distributed way. In the algorithm proposed by Luo et al. [60], each node uses the minimum standard deviation optimization (MSDO) criterion and the minimum error propagation optimization (MEPO) criterion to select reliable anchor nodes to obtain initial positions. Then, the particle filter with the Monte Carlo sampling method is utilized to calculate the final results.

#### 5.1.2. Formulation of Kalman-Filter-Based Localization

Among the filter-based methods, the most widely used is the Kalman filter. Therefore, we focus on the application of Kalman filtering and its variants in distributed relative localization of multiple robots [57]. The main steps of traditional Kalman filtering or extended Kalman filtering consist of prediction and update. Let $x_{t}$ represent the robot’s state at time *t* and $P_{t}$ represent the covariance matrix. The prediction stage is introduced as:

(13) $$\begin{array}{c} {\bar{\mu }}_{t}=g\left(u_{t},\mu_{t-1}\right)\\ {\bar{P}}_{t}=G_{t}P_{t-1}G_{t}^{T}+R_{t} \end{array}$$

where $u_{t}$ is the control model, g(·) represents the state transition function, and $G_{t}$ is the Jacobian matrix. The update stage is written as:

(14) $$\begin{array}{c} K_{t}={\bar{P}}_{t}H_{t}^{T}{\left(H_{t}{\bar{P}}_{t}H_{t}^{T}+Q_{t}\right)}^{-1}\\ \mu_{t}={\bar{\mu }}_{t}+K_{t}\left(z_{t}-h\left({\bar{\mu }}_{t}\right)\right)\\ P_{t}=\left(I-K_{t}H_{t}\right){\bar{P}}_{t} \end{array}$$

where $K_{t}$ is the Kalman gain, $z_{t}$ is the measurement, and h(·) is the observation function.

In a tightly coupled vision navigation system using Kalman filtering, the robot’s state vector contains the current state and environment feature points [61,62]. This maintenance mode of state vectors leads to higher localization accuracy and map-building quality. However, as time accumulates, or when the environment is rich in feature points, maintaining a large state vector increases the computational overhead, which affects the real-time performance.

One solution is the MSCKF (multi-state constraint Kalman filter) [63]. Multi-state constrained Kalman filtering is for VIO (visual inertial odometry) in the framework of EKF to fuse IMU and visual information. This method maintains only the current state and a history of states in a sliding window. Environmental feature points exist as constraints between robot states, thus, avoiding the dimensional explosion. MSCKF is already widely used in single-robot localization [64,65].

#### 5.1.3. Distributed Kalman-Filter-Based Relative Localization

**Distributed Kalman Filter:** Kalman filtering or its modifications are also applied in multi-robot network localization systems. The paper [66] transformed the centralized extended Kalman filter into a decentralized form. A “small” Kalman filter runs distributively on each robot. When no observation occurs between robots, each robot estimates its pose as a single robot. When two robots observe each other, they communicate to exchange states and measurements. The robots calculate the Kalman gain based on the synergy information and update their state in the update phase.

As observations are generated between robots, correlations are created between their states, and thus cross-covariances need to be tracked for state updates. The algorithm considers the correlation between the multi-robot states brought by the observations and tracks the changes. However, the algorithm requires all-to-all reliable communication. Loss of data may cause the failure of correlation tracking. At the same time, the Kalman filter dimensionality explosion problem may be more severe in systems with multiple robots.

**Distributed MSCKF and Distributed VIO:** Due to the accuracy, efficiency, and robustness of MSCKF and high-quality open-source vision odometry, such as open-VINS [67], the MSCKF framework has been used in recent years for filter-based cooperative multi-robot localization. In the distributed MSCKF, co-viewed environmental feature points can be used as observations between two robots, which is applied to the update of states and covariances [68]. In the algorithm proposed by Zhu et al. [69], IMU pre-integration is used for the prediction process of filtering.

Vision-detected feature points are classified as independent features and common features. Independent VIO without mutual observation uses independent feature points. When there are enough common feature points, the robot collects the states and covariances of its neighbors. It updates its states and covariances by triangulating the feature points to establish constraints between states. Every single robot takes the open-VINS [67] as the VIO framework.

**Distributed MSCKF Incorporating Ranging Information**: In addition to fuse vision and IMU data, the MSCKF can further utilize the ranging information between robots [70]. There may be distance measurements between robots apart from the direct target detection and common visual feature point matching. The distance measurement model is added to the observation equation in the method proposed by Chenchana et al. [71] to achieve better localization precision. However, sensors for ranging, such as UWB, can be unstable in complex environments. Particularly in the presence of occlusion, the measurement error is significant, and thus the raw range information needs to be filtered. The noise point criterion is that the covariance of the data exceeds a certain threshold.

The filtering method, especially the Kalman filter or the variants of Kalman filter, can well fuse a variety of measurement information to obtain the localization results. Particularly in the process of continuous motion, the robot can obtain smooth estimation results. However, the filter methods usually suffer from accumulated error and are highly sensitive to data-association errors.

### 5.2. Distributed Graph-Optimization-Based Multi-Sensor Fusion and Localization

Graph optimization is the representation of the optimization problem in the form of a graph. In graph theory, a graph consists of vertices and edges. Vertices represent the variables to be solved, and the edges represent the constraints between the variables. The robot network localization problem can be transformed into a nonlinear optimization problem. By designing the error of positioning as a quadratic term, the positioning problem is a least squares problem and can be written in the form of a graph optimization. The graph-optimization problem is expressed as:

(15) $$\begin{array}{cc} F(x)= & \sum_{k\in C}e_{k}{\left(x_{k},z_{k}\right)}^{T}\Omega_{k}e_{k}\left(x_{k},z_{k}\right)\\ x^{*}= & argminF(x) \end{array}$$

where $x={\left(x_{1}^{T},\ldots ,x_{n}^{T}\right)}^{T}$ is the state vector; $z_{k}$ and $\Omega_{k}$ represent, respectively, the mean and information matrix of a constraint; and $e_{k}\left(x_{k},z_{k}\right)$ is the error function.

The graph-optimization problem can be solved by numerical optimization methods, such as Gaussian Newton (GN) and Levenberg–Marquardt (LM). Due to open source graph optimization tools, such as g2o [72], ceres [73], gtsam [74], and SE-sync [75], graph optimization has been widely used in single-robot positioning [76,77] and multi-robot positioning systems [78,79].

#### 5.2.1. The Formulation of Distributed Graph Optimization

**Figure 3:**
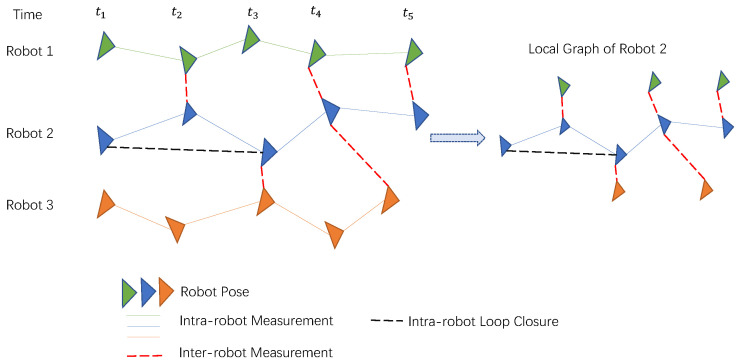
Distributed graph optimization for multi-robot localization. The left is the global pose graph, and the right figure is the local pose graph of Robot 2.

There can be multiple residual edges between the nodes of graph optimization, which provides a method for multi-sensor fusion. Graph optimization can be expressed in the form of pose graph (Figure 3). Graph optimization is also easy to parallelize or work in a distributed way by following certain rules to split the large graph into several smaller graphs.

In multi-robot distributed graph optimization, each robot node maintains and solves a local graph-optimization problem, usually centered on itself. A local graph can obtain the information from neighbors through communication in addition to the information of the center node. The optimization problem for node *i*’s local graph (similar to Equation (15)) is written as:

(16) $$\begin{array}{cc} F{\left(x\right)}_{i}= & \sum_{k\in \{i\}\cup N(i)}e_{k}{\left(x_{k},z_{k}\right)}^{T}\Omega_{k}e_{k}\left(x_{k},z_{k}\right)\\ x_{i}^{*}= & argminF_{i}\left(x\right) \end{array}$$

where N(i) represents the neighbor set of node *i*. The definition of N(i)’s content may vary in specific different algorithms and systems, depending on the communication, sensing, and computational capabilities of the robotic network.

Each constraint in the summation of Equation (16) represents an edge in graph optimization. The specific form of the edge model in graph optimization depends on the observation model of the robots. Here, we list several commonly-used edges in the graph-optimization model, taking robot node *i* as an example:

**The distance edge:** The sensors and model of distance measurements were introduced in Section 3. The formulation of distance edge is:

(17) $$e_{dis}=d_{ij}-∥X_{i}-X_{j}∥$$

where $d_{ij}$ represents the measured distance between robot *i* and *j*. $∥X_{i}-X_{j}∥$ is the distance estimated from the current states of *i* and *j*.

**The re-projection edge:** When vision-detection algorithms are available, we can detect in images landmarks or other robots whose positions are known or can be estimated. The difference between the detected pixel coordinates and the pixel coordinates obtained by reprojection is the reprojection error:

(18) $$e_{pro}=u_{ij}-project\left(X_{i},X_{j}\right)$$

where $u_{ij}$ is the observed pixel coordinates and $project(X_{i},X_{j})$ is a function to re-project the target *j* onto the pixel plane of *i*.

**The ego-motion edge:** When a single robot has an odometry system to estimate the ego-motion, we can build an ego-motion edge (also called an odometry edge):

(19) $$e_{odo}=z_{i}^{t,t+1}\left({\left(X_{i}^{t}\right)}^{-1}X_{i}^{t+1}\right)$$

where $z_{i}^{t,t+1}$ is the ego-motion result from the odometry system.

**The loop closure edge:** When the robot arrives at a scene that it has experienced before (intra-robot loop closure) or that other robots have experienced (inter-robot loop closure), the robot can obtain a new constraint by matching it with the observations in the historical data:

(20) $$e_{loop}=z_{ij}^{t_{1},t_{2}}\left({\left(X_{i}^{t_{1}}\right)}^{-1}X_{j}^{t_{2}}\right)$$

where $z_{ij}^{t_{1},t_{2}}$ is the measurement from loop closure. The value of *j* depends on the specific robot with which the loop is generated.

There are more than the listed designs of edges in graph optimization, and the form of edges may be different from the above. In the specific design of the algorithm, we often need to consider various factors, such as the robot’s sensor models and the solving process.

#### 5.2.2. Algorithms for Distributed Graph Optimization

The development of graph-optimization models is mature; however, the design of specific distributed algorithms to realize distributed graph optimization needs to consider more aspects. The considered issues include: (1) How are individual sensor data pre-processed and aligned for fusion? (2) How to optimize the solving process to avoid delays? (3) What is the scale of subgraphs according to the range of communication? These issues are considered in the literature.

In paper [78], each drone is equipped with a monocular camera, IMU, and UWB. The algorithm establishes the odometry edge by VIO, the target detection edge by visual target detection, and the range edge by UWB data. Each drone can communicate with other all drones, and each must calculate the poses of the entire network of drones, which imposes an enormous computational and communication burden.

In order to achieve real-time operation as much as possible, the method prunes the target state of the optimization. When the UAV is in the hover state, the corresponding odometer edges and distance detection edges are removed from the graph optimization, thus, reducing the computational effort of the optimization. However, since each UAV needs to calculate the entire system’s state and GPUs are required for both VIO and target detection, the algorithm has high requirements for computing power.

The authors enhanced the method in a subsequent paper [80] by adding a fisheye camera to achieve omnidirectional perception. The algorithm adds a new map-based constrained edge. The 3D point cloud map is derived from the triangulated coordinates of environmental feature points observed by multiple fisheye cameras together. To reduce the computational cost, object detection is performed in only one direction of the camera.

UWB ranging is a more direct and inexpensive measurement method among the sensors. UWB data has a high frequency and unstable characteristics. Fusing UWB ranging requires special considerations. Traditional methods are generally divided into loosely coupled [81] and tightly coupled [82]. The former means that the odometry is calculated on a separate algorithm, and the results are subsequently fused with the range information, which aggravates the long-term position drift. In tight coupling, the UWB is also involved in the joint optimization of the odometry.

Recently, researchers have considered combining the two options to avoid the shortcomings [83]. The authors provided a two-stage collaborative distributed multi-robot localization algorithm. In the short-term phase, the algorithm tightly couples odometry and ranging to obtain accurate short-term localization results. In the long-term phase, the long-term localization estimation comes from the loosely coupled fusion to reduce the computation cost. The idea of range-focus [84] was borrowed for the short-term tight-coupling phase. Using the UWB frequency as a benchmark, each round of graph optimization iterations is performed according to the UWB data cycle to better utilize more high-frequency ranging information.

The previous work usually filters the raw UWB data according to the fluctuations of the UWB data, removing the outliers. However, it is possible that the real data will be filtered out while removing the outliers, as the ranging data will change as the robot moves. Boroson et al. [85] proposed a more conservative solution. They set the ranging constraint to switchable constraints [86]. The results were evaluated after certain phases of optimization are completed. The ranging constraint is turned off when the optimization produces inconsistent results, as the ranging information may be incorrect at this point.

### 5.3. Other Solvers for Distributed Graph Optimization

The graph-optimization problem in the previous section is usually solved using the GN or LM [72,73] method. There are other solution methods that can also be used to solve distributed graph-optimization models, which may perform better in certain scenarios.

(1) *Gauss–Seidel Algorithm*

Choudhary et al. [87] proposed a two-stage trajectory estimation method based on the graph-optimization model. The algorithm assumes the existence of relative six-dimensional state measurements between robots. In the first stage, the method transforms the nonlinear optimization problem into a linear equation solving using the distributed Gauss–Seidel method. After that, each robot obtains an estimate of its rotation. In the second stage, with known rotations, the robot solves the six-DOF pose using the Gaussian–Newton method.

(2) *Non-Convex Riemannian Optimization*

Asynchronous stochastic parallel pose graph optimization (ASAPP) [12] is an asynchronous algorithm for distributed pose graph optimization in multi-robot localization. The algorithm solves the non-convex Riemannian optimization problem for pose graph optimization. Each robot executes its local optimization loop, which contains a communication thread and a computation thread. In the communication thread, robots exchange information with their surrounding neighbors. For the computation thread, the algorithm defines a Poisson clock and performs one calculation in one time period of the clock.

Each robot computes the Riemann gradient based on the local information and then computes the updated state in the negative gradient direction in a certain step. The authors also refined and improved the algorithm [88]. The new algorithm performs sparse semidefinite relaxation on the pose graph optimization and is proven globally optimal. In detail, distributed Riemannian staircase is used to perform a local search to find the gradient of error descent. Then, the solver minimizes the error function of Riemann optimization by Riemannian block coordinate descent (RBCD). The improved algorithm achieves higher accuracy and robustness.

(3) *Factor Graph Optimization*

Factor graph optimization and smoothing and mapping (SAM) [89,90] are also representations of graph optimization and are applied in robot localization and mapping. For distributed factor graph optimization, Cunningham et al. [91] addressed the problem of multi-robot distributed localization in the form of decentralized data fusion. Their algorithm aims to accomplish distributed factor graph construction and solutions. First, each robot performs a local optimization to finish SAM and to condense the local map.

To reduce communication costs, the factor graph is eliminated so that only variables associated with a neighbor are transmitted to the neighbor. The neighborhood optimization module merges the condensed local graphs into a single graph and then optimizes the graph to obtain the localization result.

However, the method does not consider the error double-counting problem. When a robot passes its measurement information to a neighbor, the measurement error will return if that neighbor has another network topology connection to this robot. DDF-sam2 [10] solves this problem by introducing an anti-factor. When the information matrix and the Jacobian matrix contain measurement information obtained from other robots, an anti-factor needs to be added at the matrix’s corresponding position to counteract the double-counted measurement factor.

In recent years, researchers have increasingly focused on the relative localization method of multi-sensor fusion and achieved better results. We compare several related systems as shown in Table 3, and there are more excellent works. Systems based on multi-sensor fusion are also typically lightweight, scaleable, and accurate. Access to data from more dimensions becomes possible. However, the multi-measurement fusion algorithm still faces challenges, such as the computational and communication burdens from more measurement information.

## 6. Distributed Simultaneous Localization and Mapping

SLAM (simultaneous localization and mapping) [92] can help robots to complete localization and mapping in an unknown environment. SLAM has become a fundamental problem for robot navigation and autonomous driving. Existing SLAM is mainly implemented by camera [93] or LIDAR [94,95] or by adding IMU for multi-sensor fusion [77,96]. SLAM is very similar to some of the previously mentioned localization algorithms.

For example, they both need to solve for the precise state of the robot, mostly using filtering and graph-optimization methods. However, the two are very different: SLAM algorithms need to build high-precision maps, and so they estimate the location of landmarks in the environment and involve techniques such as map merging. In addition, SLAM focuses more on the design of the system and less on theoretical studies than on algorithms, such as graph optimization as mentioned in the previous section.

Collaborative SLAM means that multiple robots work together through communication, observation, and other collaborative means to complete positioning and mapping [97,98]. Collaborative SLAM, as an enhancement to single-robot SLAM, has broader application scenarios, such as VR or AR [99]. The framework of collaborative SLAM can be roughly divided into centralized, server-client-based, and distributed. The centralized SLAM algorithm centralizes all data to one robot for computation [100].

In the server-client architecture, the robots’ data are transferred back to the server, which has powerful computing and communication bandwidth [101,102]. In distributed SLAM, each robot performs localization and map building, cooperating with each other but without a centralized machine [103,104].

### 6.1. Distributed SLAM Algorithms

Distributed collaborative SLAM incorporates the task of multi-robot collaboration. At the same time, the tasks of localization and map building are decentralized to each robot for execution [105]. Distributed cooperative localization is a fundamental part of cooperative SLAM. It is also necessary to give a brief overview of distributed cooperative SLAM. This survey focuses on distributed and decentralized algorithms in this section.

#### 6.1.1. Distributed Loop Closures

Cieslewski et al. [106] proposed a decentralized visual SLAM method with efficient data association. Each robot calculates the full image descriptor by NetVLAD [107]. The visual descriptor and other information for relative estimation are only sent to one robot for communication efficiency. The whole system takes ORB-SLAM as a visual odometry framework. The back-end solving process is changed to solve the rotation matrix first and complete the 6-D pose later.

However, visual loop closure detection as mutual observation between robots produces erroneous observations in areas with weak literature, low light, and similar scenes. Addressing the loop closure problem, Lajoie et al. presented DOOR-SLAM [108]. The visual front-end and feature extraction network is similar to paper [106]. Due to observation noise and errors in feature point extraction and matching, loop closure detection may be incorrect. The algorithm uses PCM [109] to determine the loop closure outliers in the distributed outlier rejection submodule. For the distributed graph optimization submodule, the authors utilized the distributed Gauss–Seidel algorithm [87] to calculate the trajectory of each robot.

LIDAR is not affected by light and texture and uses a special feature extraction scheme. For DiSCo-SLAM [110], the authors used the lightweight Scan Context descriptor [111] to reduce the communication burden of information exchange between robots, improving the accuracy and efficiency of distributed loop closures.

#### 6.1.2. Relocalization and Global Localization

Relocalization is to retrieve the position of the robot’s current frame in the world frame when the robot’s tracking is lost. In multi-robot SLAM, the lost robot can obtain the localization information again with the cooperation of other robots [112]. Specifically, the relocalization module matches the data of the robot’s current frame with the map frame or other robots’ data frames within a certain range. This process is similar to the loop closure mentioned above, which can also be implemented using the bag-of-words model [113] or NetVLAD [107].

Global localization locates the position of the current robot in the existing map. In dynamic multi-robot tasks, global localization is of great significance and can enable newly added robots to quickly initialize their states. In addition, robots that have been disconnected for a long time due to unexpected errors can also obtain their location through global localization and rejoin the swarm.

#### 6.1.3. Data Association

Data association is the integration of data collected by the SLAM front-end to obtain the final localization results. The data collected by the front end include vision, inertial measurement, wheel speed, loop closures, relocalization, and global localization information. Data association is usually completed at the back end, and the specific method is similar to multiple measurement fusion algorithms (Section 5), including particle filters [114], Kalman filters [115], graph optimization [116], Gauss–Seidel [117], and so on.

### 6.2. Distributed Mapping-Oriented Algorithms

Robots usually need to exchange maps during collaboration, which can impose a significant communication burden. Communication immediacy is extremely important for distributed SLAM. Downsampling the map reduces the communication cost but reduces the quality of the mapping. One way to improve communication while maintaining map quality, for example, is to change the form of map management. Choudhary et al. [118] propose an object-based SLAM with optimized map representation.

The algorithm first performs object detection on RGBD images using a convolutional neural network. Then, segmentation of the object and estimation of its pose is conducted. The segmented instance is added to the map as a landmark. Object-based maps are significantly better than feature-based maps in storage and communication. However, this method requires advanced training of network parameters and finds it more challenging to handle unfamiliar objects in the environment.

High-quality maps are essential for robots to perform navigation, path planning, and other execution tasks. Dense maps have more information compared with sparse feature maps. Single robots have a limited view of map building, so multi-robot collaborative dense mapping has better prospects.

**Figure 4:**
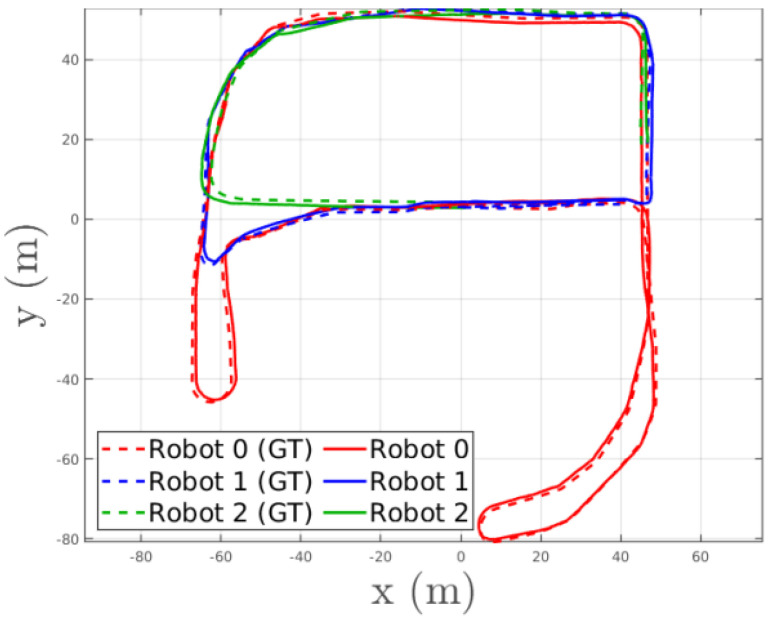
Estimated trajectories of Kimera-Multi [119] compared to the ground truth (GT). The absolute trajectory error (ATE) in that experiment was 1.46 m.

Yun et al. [119] proposed Kimera-Multi, a system for distributed multi-robot metric-semantic simultaneous localization and mapping(Figure 4). A team of robots is equipped with visual-inertial sensors, and each robot runs a local pose estimation (Kimera-VIO) and local mesh using Kimera [120]. When two robots are in communication range, they perform distributed place recognition to obtain observations between states and perform collaborative pose graph optimization. In this process, the poses and maps of the two robots are solved optimally.

We compare serveral distributed SLAM works in Table 4. Overall, some initial developments have been made in distributed collaborative SLAM. Traditional SLAM solutions for single robots have been improved to be used in multi-robot collaboration scenarios. However, some issues are worth exploring in-depth, such as the communication problem of large-scale clusters, the adaptive ability of dynamic scenes [98], and the limited computing power of edge devices. From merely completing the localization task to both localization and mapping, more theoretical and engineering consideration is needed.

## 7. Local Subnetwork Organization and Communication

### 7.1. Local Subnetwork Organization

In distributed localization, each node in the network needs to aggregate the known neighbor information before computing. A node will form a local subnetwork with its neighboring nodes. The subnetwork size depends on the range of communication and the sense of the nodes. The larger the sub-network, the richer the local information is and the higher the accuracy of the distributed algorithm. However, the size of the sub-network needs to require the nodes to have the corresponding communication bandwidth and computing power.

An extreme case is that each node has the capability of network-wide communication, i.e., each node holds global information [78], which is called the decentralized architecture. However, this architecture is difficult to implement in large-scale clusters. In most works, distributed nodes have only a limited and fixed communication range and rely on limited neighbor information to perform their calculations [11,87].

### 7.2. Communication Efficiency

The nodes in the network exchange information through communication to collaborate in a distributed system. To meet the real-time nature of localization, the robot must continuously update and send its information while receiving content transmitted by its neighbors. Delayed, inefficient, or erroneous communication can lead to inaccurate localization results. The design of the communication module of the distributed algorithm is particularly important [123].

Each node can maintain a cache to store local information [12]. Each node periodically sends its information (e.g., status and observations) to its neighbors, which is used to update the local information of neighbors. At the same time, nodes also replace the corresponding entities in the cache while receiving communication messages.

One way to improve the efficiency of communication is to compress or improve the content of the transmission. In DDF-sam [91], the robots share condensed graphs, which come from marginalizing out variables from a probabilistic model. Condensed graphs save on communication overhead compared to the original graph. In the distributed SLAM algorithm based on the object model [118], the object-based communication model reduces the communication cost because the robots avoid transmitting raw sensor data.

Communication efficiency can also be improved by designing new communication protocols or scheduling strategies [124]. Cheng et al. [123] proposed a distributed estimation scheme using the Fisher information matrix (DEF), which only requires one round of communication between edge UAVs and the center UAV. In the paper [125], the authors presented a communication-efficient FedAvg method for federated indoor localization. They proposed a novel layer-wise asynchronous aggregation strategy to reduce the communication traffic during the training process.

## 8. Robust Distributed Localization

The robustness of the distributed localization algorithm is enhanced compared to the central one, and there is no need to worry about the central node’s failure leading to the whole network’s failure. However, distributed algorithms also have new challenges in robust performance, such as initialization, the dynamic topology network, and measurement noise.

### 8.1. Initialization

In the initialization phase of the distributed localization algorithm, the nodes need to be involved in network access, observation of module initialization, and localization result initialization. Among these, the localization result initialization is particularly important. A better initial localization value can accelerate the subsequent algorithm’s convergence [117].

An easy-to-implement solution is called flagged initialization [87]. Nodes that have completed initialization are marked. Nodes that still need to complete initialization rely on initialized neighbors. Another similar method is to spread the measurement and other information in the network in the order of a spanning tree [12]. However, in some extreme cases, some nodes in the network may be unobservable, or the initialization results may not be accurate enough. Then, random states for initialization are utilized. The initialization results are solved multiple times, and finally we can choose the best one [80].

### 8.2. Measurement Noises

Due to factors such as the sensor manufacturing process and environmental interference, the measured value is usually noisy. Measurement noise will affect the accuracy of the location. The algorithm, without considering measurement noise, is difficult to apply to the real world.

The measurement error can significantly affect the localization results in the distance-only distributed algorithm. These errors can be resolved by transforming the closed-form solution into an optimal solution [29]. In addition, the solution process can be relaxed to avoid the difficulty of unsolvability due to noise [26]. In the multi-sensor fusion mode, the effects of errors can be reduced due to the presence of multiple sensors. For example, ranging can correct the cumulative drift of VIO.

## 9. Challenges and Future Study on Distributed Localization

The research of robot clusters has made extensive progress since the last century. The localization technology of robot clusters, including centralized and distributed, global localization, and relative localization, is a basic technology in theory and application. Distributed relative localization has strong advantages, such as scalabilty and robustness, which has attracted researchers’ great interest. However, due to the limitations of hardware equipment or theoretical research, there are still some challenges in the current research of distributed relative localization. In this section, we will introduce the main challenges and future research trends.

### 9.1. Dynamic Topology

In practical applications, the topological links of robot networks may be dynamic. For example, new UAVs may join the UAV swarm at any time. Therefore, the localization algorithm must adapt to the dynamic topology of the network. In the Omni-swarm proposed by Xu et al. [80], the “Plug-and-Play” strategy allows the temporary join or exit of the drones by quickly initializing the new nodes in the network. However, if the newly added robot(s) has a large amount of trajectories or map points, the problem will be transformed into topology splicing or healing [126]—that is, the two networks need to be merged.

### 9.2. Scalability

Most of the existing studies have used small-scale robot clusters as their research objectives. In practical tasks, large-scale clusters can often undertake more tasks and cover a wider area [127]. This will be a challenge for distributed relative localization algorithm to undertake large-scale clusters (such as more than 100 robots). Specifically, how can a robot with only local perception obtain global information? How can the communication protocol ensure no conflict or less conflict? How dense should the network be?

### 9.3. Distributed Active Localization

Among the algorithms discussed in this survey, the majority of robot localization algorithms are “passive”—that is, the localization algorithm will not affect the control and real state of the robot. When the localization algorithm degrades or the error increases continuously, the robot cannot correct it in time by adjusting itself [128]. On the contrary, in active localization, the robot can adjust its pose through the control system so that it can obtain more measurement information or more accurate positioning results [129].

## 10. Simulation Platforms and Datasets

The rapid development of technologies, such as robotics, autonomous driving, drones, and the Internet of Things, has attracted the interest of many researchers. In the research process, the proposal of new technologies requires experimental verification, and thus there is a considerable demand for the construction of experimental platforms.

However, devices such as robots, drones, and unmanned vehicles are often costly and challenging to deploy. Performing real-world experiments without validated algorithms is risky for both the experimenters and equipment. Therefore, verifying the system or algorithm on the simulation platform is essential.

In this work, we present a brief survey on the simulation platform for the multi-robot experiment. We compared several simulation platforms in the following aspects:

- Open source. The open source experimental platform allows researchers to change the details of the simulation according to their experimental needs, such as the model of the robot, the type and parameters of the sensors, and the communication mode. The open source platform has better scalability.
- Supported OS. Windows, Linux, and Mac are the most popular operating systems on the market. Robots or other edge devices may run on any of these operating systems. Therefore, a simulation platform adapted to these operating system is closer to the real world.
- ROS supported. Robot Operating System (ROS) [130] is one of the most popular robot software frameworks. The simulation platform can provide data sources and operational feedback for ROS to simulate real-world experiments.
- Model. The model of the simulation object is the most critical part of the experiment. The model here includes not only the physical appearance but also sensors, communication modules, and computing power.
- Scene quality. The robot’s ability to perceive and operate on the environment is basic. Therefore, the quality of the environment should be as close to reality as possible and also simulate the noise in the environment.
- Distributed capabilities. Simulation experiments are usually executed on a single machine; however, the multi-robot distributed localization algorithm is designed to be executed on multiple robots in a distributed manner. This is also important for the implementation of distributed simulation.

The comparison of different platforms based on the aspects above is presented in Table 5. In particular, most simulation platforms do not support distributed simulation, which is detrimental to the simulation verification of multi-robot distributed collaborative algorithms. Researchers need to design their own distributed simulation protocols. We hope that, in the future, there will be more simulation platforms that support distributed capabilities.

## 11. Conclusions

In this work, we analyzed and summarized distributed localization algorithms for multi-robot networks. We analyzed the characteristics and limitations of different distributed algorithms. We also introduced the distributed SLAM problems, which emerged in recent years. Then, we explored the communication efficiency, local network organization, and robustness that need to be considered in distributed localization algorithms. The challenges and trends on this research were also discussed in this survey. Finally, we surveyed existing simulation experimental platforms in the hope that we could provide a reference for other researchers to conduct simulation experiments. We propose that large-scale multi-robot networks will be a huge trend in the future. Efficiently solving the state of each robot in the network is a necessary task.

## Figures and Tables

**Table 1:** Comparison with related surveys.

Survey	Date	Focus	Multi-Robot Localization	Distributed Methods	Challenges & Trend	Experiment Platform
[15]	2014	Collaboration systems	Yes	Few	No	No
[16]	2016	Localization	Yes	Few	Yes	No
[17]	2019	Localization, IoT	Yes	Medium	Yes	No
[18]	2020	Localization, UWB	Yes	Few	No	No
[14]	2021	Localization, single robot	No	No	Yes	No
[21]	2021	Localization, single robot	No	No	Yes	No
[20]	2022	SLAM	Yes	Few	Yes	No
Our work	2022	Distributed relative localization	Yes	Most	Yes	Yes

**Table 2:** Comparison of distributed location algorithms based on distance measurement. Note that *s* is a parameter set in advance, which may be different in different papers.

Algorithm Category	Paper	Anchor Number	Characteristics
Barycentric Coordinate-Based	[24,27,29]	≥3	Slow convergence speed; Sensitive to noise; Topology may be limited to convex hull.
Distributed MDS	[9,33,50]	≥0	Dense network; Sensitive to noise.
Distributed SDP	[41,43,49]	≥0	Dense network; Sensitive to noise; High convergence speed.
Nonlinear Optimization	[11,46]	≥s	Tolerate to sparsity in some extend; High convergence speed; General model.

**Table 3:** Comparison of several multi-measurement fusion works.

Solution	Year	Method	Sensors	Characteristics
Graph optimization	2020	Xu et al. [78]	Camera, IMU, UWB	Involing range measurement. Decentralized scheme.
	2022	Omni-swarm [80]	Fisheye-camera, IMU, UWB	Omni-directional.
	2022	Nguyen et al. [83]	Camera, IMU, UWB	Range-focused fusion.
Filter	2018	Chenchana et al. [71]	Camera, IMU, UWB	Involving range measurement
	2021	DISC-VIO [69]	Camera, IMU	Using MSCKF. Only simulation experiment.
Factor graph	2013	DDF-sam [10]	Camera, IMU, GPS	Factor graph. Anti-factor.
Gauss–Seidel	2016	Choudhary et al. [87]	LIDAR, IMU, wheel odometry	Two-stage distributed Gauss–Seidel approach.

**Table 4:** Comparison of several distributed SLAM works.

Year	Method	Sensors (Frontend)	Backend	Characteristics
2017	Choudhary et al. [118]	LIDAR, IMU, wheel odometry	Gauss–Seidel	Object-based model
2018	Cieslewski et al. [106]	Camera	Gauss–Seidel	Data efficient.
2020	DOOR-SLAM [108]	Camera	Graph optimization	Outlier rejection. Communication Efficiency.
2021	Karrer et al. [121]	Camera, IMU, UWB	Filter	Invole range measurement
2022	Kimera-multi [122]	Camera, IMU	Graph optimization	Semantic map. Outlier Rejection.

**Table 5:** Comparison between simulation platforms.

Platform	Open Source	Supported OS	ROS Supported	Model	Scene Quality	Distributed Capabilities
AirSim [131]	✓	Win, Linux	✓	Car, Quadrotor	High	
Gazebo [132]	✓	Linux, MacOS	✓	Robot, Quadrotor	Middle	
RflySim [133]	✓	Win	✓	Car, Quadrotor, Fixed-wing	High	✓
Jmavsim [134]	✓	Win, Linux, MacOS	✓	Quadrotor	Low	
Morse [135]	✓	Linux	✓	Robot, Quadrotor	Middle	
FightGear [136]	✓	Win, Linux, MacOS		Quadrotor, Fixed-wing	Low	
XPlane [137]	✓	Win, Linux		Fixed-wing	High	
HackFlightSim [138]	✓	Win, Linux	✓	Fixed-wing, Multirotor	Middle	

## Data Availability

No new data were created.
